# Cutaneous Human Papillomavirus Infection and Development of Subsequent Squamous Cell Carcinoma of the Skin

**DOI:** 10.1155/2016/1368103

**Published:** 2016-11-07

**Authors:** Shalaka S. Hampras, Rhianna A. Reed, Spencer Bezalel, Michael Cameron, Basil Cherpelis, Neil Fenske, Vernon K. Sondak, Jane Messina, Massimo Tommasino, Tarik Gheit, Dana E. Rollison

**Affiliations:** ^1^Department of Cancer Epidemiology, Moffitt Cancer Center, Tampa, Florida, USA; ^2^University of South Florida, Morsani College of Medicine, Tampa, Florida, USA; ^3^Department of Dermatology, University of South Florida, College of Medicine, Tampa, FL, USA; ^4^Department of Cutaneous Surgery, University of South Florida, College of Medicine, Tampa, FL, USA; ^5^Cutaneous Oncology Program, Moffitt Cancer Center, Tampa, Florida, USA; ^6^Departments of Pathology and Cell Biology, University of South Florida, College of Medicine, Tampa, FL, USA; ^7^Infections and Cancer Biology Group, International Agency for Research on Cancer-World Health Organization, Lyon 69372, France

## Abstract

The role of cutaneous human papillomavirus (HPV) infection in the development of subsequent cutaneous squamous cell carcinoma (SCC) is unknown. Pathologically confirmed cases of SCC (*n* = 150) enrolled in a previously conducted case-control study were included in a retrospective cohort study to examine the association of cutaneous HPV at the time of SCC diagnosis with the risk of subsequent SCC development. Data on HPV seropositivity, HPV DNA in eyebrow hairs (EB) and SCC tumors were available from the parent study. Incidence of subsequent SCC was estimated using person-years of follow up. Cox Proportional Hazards ratios were estimated to evaluate the associations of both, HPV seropositivity and HPV DNA positivity with subsequent SCC. The five year cumulative incidence of subsequent SCC was 72%. Seropositivity to cutaneous HPV was not associated with the risk of subsequent SCC (HR = 0.83, 95% CI = 0.41–1.67). Any beta HPV infection in EB was associated with reduced risk (HR = 0.30, 95% CI = 0.11–0.78) of subsequent SCC among cases who were positive for beta HPV DNA in tumor tissue. Infection with beta HPV type 2 (HR = 0.32, 95% CI = 0.12–0.86) in EB was associated with reduced risk of subsequent SCC among HPV DNA positive SCCs. In conclusion, beta HPV infection was inversely associated with the risk of subsequent SCC.

## 1. Introduction

Keratinocyte cancer (KC), including squamous cell carcinoma (SCC) and basal cell carcinoma (BCC), is the most commonly diagnosed cancer in the United States [1]. While ultraviolet radiation is an established risk factor for KC [2], growing evidence suggests infection with cutaneous human papillomavirus (HPV) may increase the risk of cutaneous SCC [3–8]. We previously reported an increased risk of SCC associated with the presence of four or more types of cutaneous HPV DNA in eyebrow hairs [3]. Serological responses to genus beta HPV types were found to be associated with increased risk of SCC [4]. Further, poor tanning ability was associated with 6.9 times increased risk of SCC among those who were seropositive to beta HPV type [5]. Collectively, these findings suggest that cutaneous HPV may play a role in the pathogenesis of SCC. However, it should be noted that some previous case-control studies reporting an association between beta HPV and SCC included only primary cases of SCC [6, 9], while others [3, 4] did not differentiate between primary and subsequent SCCs. Therefore, the role of cutaneous HPV infection in the development of subsequent SCC is unknown.

As compared to BCCs, SCCs are more likely to be clinically aggressive with a propensity to recur and metastasize [1, 10]. As reviewed previously, patients with metastatic SCC have less than 20% survival rate over 10 years [1]. Varying recurrence rates for SCC have been reported based on the site of involvement, metastasis, and grade. In a retrospective study, 30% SCCs involving temporal bone were found to recur within an average of 5.8 months [11]. Others have reported lower SCC recurrence rates of 9–15% [12, 13]. As summarized by Alam and Ratner, risk factors for recurrence of SCCs include tumor size, site, tumor depth, perineural invasion, history of treatment for SCC, and tumor differentiation [1]. While these factors may aid in the identification of SCC cases at high risk of recurrence, they provide limited scope for intervention beyond treatment or close surveillance to prevent the recurrence of SCC.

SCC cases are also at a high risk of developing second primary SCCs. In a large population based study of over 25,000 Swedish SCC cases, a significantly increased risk of second primary SCC was observed (standardized incidence ratio = 15.6) [14]. Meta-analyses results have shown that the risk of developing SCC subsequent to an “index” SCC was 18% [15]. As cautioned by Marcil and Stern, some of the previous studies examining the risk of a subsequent SCC included index SCC cases with a history of >1 SCC or did not provide such information. Thus, the index SCCs may have comprised both first and recurrent SCCs [15]. The high morbidity associated with development of subsequent SCCs warrants further research.

Given the increased risk of SCC observed in association with cutaneous HPV infection as described above, it is reasonable to hypothesize that patients with SCCs associated with cutaneous HPV infection are more likely to develop subsequent SCCs compared to patients with SCCs that are not associated with HPV.

Using a subset of SCC cases (*n* = 150) from our previously conducted study [3, 4], we conducted a retrospective cohort study to examine the association between cutaneous HPV infection at the time of SCC diagnosis in the parent study with the risk of subsequent SCC. Infection with cutaneous HPV was assessed using three biomarkers: serum antibodies to HPV, presence of HPV DNA in eyebrow hairs, and presence of HPV DNA in SCC tumors.

## 2. Materials and Methods

### 2.1. Study Design and Population

The study population has been described previously [3, 4, 16]. Briefly, a clinic based case-control study was conducted at Moffitt Cancer Center in Tampa, Florida, in 2007–2009. Histologically confirmed cases of SCC (*n* = 173) were identified through the University of South Florida (USF) Dermatology Clinic. Both newly diagnosed primary SCCs and cases with a prior history of SCC were included in the case group. Controls included patients without history of any type of cancer and who were negative for skin cancer based on a full-body screening exam at the time of enrollment. Of the 173 SCC cases enrolled in the parent case-control study, 150 SCC cases were followed through 2014. The remaining cases either did not return to the clinic or were only referred to USF Dermatology Clinic for Moh's surgery. Cases without complete follow-up were similar in age and gender distribution to the cases with complete follow-up. The 150 “index” SCC cases with complete follow-up through 2014 were included in the present analyses. Serology data on 33 cutaneous HPV types, including HPV types in the alpha, beta, and gamma genera (*n* = 150), as well as beta HPV DNA (*n* = 25 beta HPV types) status in eyebrow hairs (*n* = 145) and SCC tumor tissue (*n* = 131), at the time of enrollment of index SCC, were available from the parent study [3, 4]. Self-reported data on demographics, smoking, alcohol consumption, and skin cancer risk factors, including history of blistering sunburns, sun exposure, and tanning ability were also available from the parent study. For the current retrospective cohort, subsequent SCC was defined as the SCC, including recurrent and second primary SCC, detected after the index SCC was diagnosed at the enrollment biopsy in the parent case-control study [3, 4]. Medical charts of index SCCs were reviewed, and data on the date of past history of SCC prior to enrollment of an index SCC case in the parent case-control study, date of subsequent SCC diagnosed after the index SCC enrollment, site and tumor dimensions of subsequent SCC, other incident keratinocyte cancers and date of last contact or death were abstracted. All participants provided written informed consent, and the study protocol was approved by the Institutional Review Board at USF.

### 2.2. Serology

Serum samples obtained from the index SCC cases at the time of their enrollment in the parent study were examined for antibodies to major capsid protein L1 for 33 cutaneous HPV types including genera alpha (2, 3, 7, 10, 27, 57, and 77); beta [beta 1 (5, 8, 20, 24, 36), beta 2 (9, 15, 17, 23, 38, 107), beta 3 (49, 75, 76), beta 4 (92), and beta 5 (96)], gamma (4, 48, 50, 65, 88, 95, 101, 103), mu (1), and nu (41). As described previously [4], antibodies were detected using an enzyme linked immunosorbent assay [17, 18] and fluorescent bead-based multiplex serology [19].

### 2.3. HPV DNA in Eyebrow Hairs and SCC Tumor Tissue

Measurement of HPV DNA in eyebrow hairs and fresh-frozen SCC tumors has been described in detail previously [3, 4]. Briefly, HPV DNA was extracted from eyebrow hairs and SCC tumor tissue with the QIAGEN EZ1 DNA Tissue Kit, and HPV genotyping was performed using a type specific multiplex assay detecting DNA from 25 genus beta HPV types (5, 8, 9, 12, 14, 15, 17, 19, 20, 21, 22, 23, 24, 25, 36, 37, 38, 47, 49, 75, 76, 80, 92, 93, and 96) [20, 21].

### 2.4. Statistical Analyses

Follow-up time was defined as the time interval between the date of index SCC biopsy at enrollment in the parent study and date of diagnosis of subsequent SCC or date of last contact. Five-year cumulative incidence of subsequent SCC was estimated using person-years of follow-up. Demographic, lifestyle factors, and skin cancer risk factors were compared between those who developed a subsequent SCC and those who did not, using Chi Square and Fisher's exact tests, as appropriate. Cox Proportional Hazards ratios (HR) and corresponding 95% confidence intervals (CIs) were estimated for associations of seropositivity to cutaneous HPV, overall and by genera, with the risk of subsequent SCC, adjusting for age and gender. Seropositivity to “any” HPV was defined as proportions of SCC cases that were positive for an antibody to at least one HPV type across all genera (overall cutaneous HPV seropositivity), within the specific genera (genus specific seropositivity) or within specific species (species-specific seropositivity). Seropositivity to multiple HPV infections was defined as the presence of antibodies corresponding to ≥2 beta HPV types.

“Any” beta HPV infection in eyebrow hairs, overall and by species, was defined as the proportion of cases who had DNA corresponding to at least one HPV type in genus beta or in a given species within genus beta, respectively. Multiple HPV infections were defined as the presence of HPV DNA corresponding to ≥2 beta HPV types. Since SCC tumors have lower viral DNA load compared to eyebrow hairs [3], HPV DNA status in tumors was categorized as “negative for all of the 25 types of beta HPV DNA” or “positive for ≥1 type of beta HPV DNA” or “positive for ≥1 species-specific beta HPV DNA.” Cox Proportional Hazards ratios and corresponding 95% CIs were estimated for associations of beta HPV DNA positivity, overall and by species, with the risk of subsequent SCC, adjusting for age and gender. Associations of both beta HPV seropositivity and beta HPV DNA positivity in eyebrow hairs with the risk of subsequent SCC were stratified by SCC tumor beta HPV DNA status.

## 3. Results and Discussion

As seen in Table 1, SCC cases included in this study were all White, with an average age at index SCC diagnosis of 64.5 years, with 92.8% cases reporting a prior history of SCC. Of the 150 index SCC cases, 105 (70%) were diagnosed with at least one KC during follow-up. Ninety-one out of the 150 SCC cases were found to have at least one subsequent SCC since their enrollment in the previous case-control study [3, 4]. Up to five SCC tumors per case, the majority in the head and neck region, were detected at the time of diagnosis of subsequent SCC. The median follow-up time was 1.15 years. The five-year cumulative incidence of subsequent SCC was 72%.

Subsequent SCCs were detected within an average of 1 year from the date of SCC biopsy at enrollment visit. SCC cases with a past history of SCC or BCC or both were significantly more likely to have a subsequent SCC (Table 2). None of the other demographic or lifestyle characteristics were associated with subsequent SCC (Table 2). Seropositivity to cutaneous HPV infections, overall, by genera and beta HPV species, was not associated with the risk of subsequent SCC (Table 3).

Presence of beta HPV DNA in eyebrow hairs was not associated with the risk of subsequent SCC, overall or by species (Table 4). However, after stratification by tumor beta HPV DNA, “any” beta HPV infection in eyebrow hairs was significantly associated with 70% reduced risk of subsequent SCC among cases who were positive for any beta HPV DNA in tumor tissue (Table 5). Further, species-specific analyses showed that “any” beta-2 HPV infection in eyebrow hairs was associated with 68% significantly reduced risk of subsequent SCC among tumor DNA positive index SCC cases (Table 5). Beta HPV seropositivity was not associated with subsequent SCC after stratification by tumor beta HPV DNA status (Table 5).

Cutaneous SCCs contribute substantial morbidity due their tendency to recur [1] and associated high risk of development of second primary malignances of the skin and other organs [14]. With the rising incidence of SCC [22], the prevalence of recurrent and second primary SCCs will also increase contributing substantially to public health burden. It is important to identify factors predisposing to recurrent and second primary SCCs to guide development of better follow up protocols, characterize high risk SCCs, and inform preventive strategies aimed at reducing the morbidity associated with subsequent SCCs.

In this retrospective cohort study, while no association was observed between cutaneous HPV seropositivity and the risk of subsequent SCC, a significantly inverse association was observed between cutaneous beta HPV infection in eyebrow hairs detected at the time of index SCC diagnosis and subsequent SCC development, among SCC cases with HPV DNA positive index SCC tumors. These findings are in contrast to the positive association reported between cutaneous HPV infection, measured by HPV serology and presence of HPV in eyebrow hairs, and SCC [3, 4]. However, our findings are consistent with findings from previous studies on mucosal HPV infection associated cancers. Mucosal HPV infection has been previously associated with improved survival [23–25] or reduced rate of recurrence [26] in some cancers. For example, in a small study of 41 cases, recurrence rate of nasopharyngeal carcinoma was 75% in HPV tumor DNA negative patients compared to 11% in tumor DNA positive patients [26]. Ritchie et al. reported that HPV positive oropharyngeal cancers had 70% significantly improved survival compared to HPV negative cases, with significant survival benefits for men versus women [25]. As discussed previously [25], increased sensitivity to radiation treatment and reduced occurrence of P53 mutations in HPV tumor positive cases have been suggested as possible mechanisms of improved prognosis in association with mucosal HPV infection.

In the context of cutaneous SCC, which is mainly treated by excision, it is not clear whether the underlying mechanisms of improved prognosis of HPV positive SCCs are similar to those suggested for prognosis of mucosal HPV associated cancers. Cutaneous HPV infection is thought to be involved in the initiation, but not the maintenance of SCC [27, 28]. One explanation of the observed inverse association between beta HPV infection and subsequent SCC could be that beta HPV infection in index SCC tumors may be immunogenic and could potentially stimulate host immune response that prevents the development of subsequent SCCs.

Our findings may suggest different mechanisms driving tumor progression in HPV DNA positive versus HPV DNA negative SCC cases. Immunological and genetic factors that are associated with HPV DNA negative tumors should be explored further. Previously, we showed that, among a subset of SCC cases included in the present study, single nucleotide polymorphisms (SNPs) in epidermodysplasia verruciformis genes, regulating immune response against HPV, were associated with reduced risk of beta HPV DNA positive SCC tumors [29]. These SNPs should be explored further in association with the risk of subsequent SCC.

The findings from our study should be interpreted with caution. A majority (92.8%) of index SCC cases had a history of SCC prior to their enrollment in the parent case-control study. Thus, external validity of our findings may be limited to those cases with a past history of SCC. Cutaneous HPV infection status at the diagnosis of past SCCs was unknown. Therefore, association of change in HPV infection status over time with the risk of subsequent SCCs could not be evaluated. HPV DNA status in subsequent SCC tumors detected during follow-up was not measured. Due to limited sample size, robust statistical analyses for HPV type specific association with subsequent SCC development could not be conducted. The subsequent SCCs detected in this study could be recurrent SCCs, presenting at the same anatomic site as primary SCC, or second primary SCCs and the analyses stratified by these two outcomes were not conducted due to small sample. Conversely, combining recurrent and second primary SCCs as one outcome may have attenuated the true effect of HPV infection in index SCC on the development of recurrent or second primary SCCs.

The study has several strengths. To our knowledge, this is the first prospective study reporting an association between cutaneous HPV infection in SCC cases and subsequent SCCs. Of the 173 SCC cases enrolled in the parent case-control study, 86.7% cases were included in the present analyses highlighting complete follow-up on a large proportion of the cases. Since 92.8% SCC cases reported a prior history of SCC, the findings from our study may help further prognostic stratification of these SCCs using a biomarker of infection. These cases may represent ideal candidates to direct infection related prevention measures due to the high morbidity associated with risk of developing subsequent SCCs among SCCs. Based on the observed inverse association between HPV seropositivity and risk of subsequent SCC (Table 3), although not significant, immune response against HPV may protect against the development of subsequent SCCs. Thus, acquired immunity through vaccination against cutaneous HPV could potentially be explored among SCC cases. Additional strengths of this study include, examination of biomarkers of both, present HPV infection (eyebrow hair DNA and HPV serology) and past HPV infection (HPV serology) and evaluation of HPV infection as a prognostic marker by HPV genera and species.

## 4. Conclusion

In conclusion, the results from our prospective cohort study suggest that cutaneous HPV infection in eyebrow hairs is inversely associated with the development of subsequent SCCs in a subset of SCC cases with HPV DNA positive tumors. The observed association supports previous reports of lack of a role for cutaneous HPV infection in progression of SCCs. While cutaneous HPV may not be directly involved in the prognosis of SCC, this line of research may eventually lead to discovery of underlying immunological factors involved in SCC development and progression. Cutaneous HPV infection status could potentially be used as a biomarker for prognostic stratification of SCCs.

## Figures and Tables

**Table 1:** Demographic and baseline characteristics of 150 cutaneous squamous cell carcinoma (SCC) cases.

Variable	*n* (%)
*Age in years [Mean (std)]*	64.4 (10.20)
*Gender*	
Male	100 (66.7)
Female	50 (33.3)
*Race*	
White	134 (100.0)
Other	0 (0)
*Education*	
≤12th grade	23 (17.4)
>12th grade	109 (82.6)
*Skin color*	
Fair white	59 (44.4)
Medium white	69 (51.9)
Light brown	5 (3.8)
*Skin's reaction to first time exposure in the sun*	
A blistering sunburn	21 (16.0)
A sunburn without blisters	58 (44.3)
A mild sunburn that becomes a tan	35 (26.7)
A tan with no sunburn	164 (10.7)
No change in skin color	3 (2.3)
*History of blistering sunburn prior to index SCC diagnosis*	
No	29 (22.3)
Yes	101 (77.7)
*Skin's reaction to repeated exposure in the sun*	
Unable to tan	243 (17.6)
It can tan if you work at it	61 (46.6)
It tans easily	47 (35.9)
*Job in sun for more than 3 months at any time in life prior to the index SCC diagnosis*	
No	70 (53.0)
Yes	62 (47.0)
*Number of moles on the entire body*	
None	47 (35.3)
Less than 10 moles	62 (46.6)
10–25 moles	21 (15.8)
More than 25 moles	3 (2.3)
*History of smoking*	
Never	40 (30.8)
Ever	90 (69.2)
*History of SCC*	
No	8 (7.2)
Yes	103 (92.8)

**Table 2:** Associations between baseline skin cancer risk factors and subsequent cutaneous squamous cell carcinoma (SCC).

Variable	No subsequent SCC (*n* = 59) *n* (%)	Subsequent SCC (*n* = 91) *n* (%)	*P* value for Chi Square test
*Age [mean (STD)]*	62.9 (12.4)	65.4 (8.4)	0.16^1^
*Gender*			
Female	24 (40.7)	26 (28.6)	0.12
Male	35 (59.3)	65 (71.4)	
*History of blistering sunburn prior to index SCC diagnosis*			
No	15 (28.3)	14 (18.2)	
Yes	38 (71.7)	63 (81.8)	0.17
*Tanning ability*			
No	11 (21.6)	12 (15.0)	
Yes	40 (78.4)	68 (85.0)	0.33
*Job in sun for more than 3 months at any time in life prior to the index SCC diagnosis*			
No	31 (59.6)	39 (48.7)	0.22
Yes	21 (40.4)	41 (51.2)	
*Smoking*			
Never	17 (32.1)	23 (29.9)	
Ever	36 (67.9)	54 (70.1)	0.79
*Alcohol*			
Never drinker	12 (23.5)	15 (18.7)	0.51
1 or more drinks in past 1 year	39 (76.5)	65 (81.2)	
*History of SCC prior to index SCC diagnosis*			
No	7 (15.6)	1 (1.5)	
Yes	38 (84.4)	65 (98.5)	0.007^2^
*History of BCC prior to index SCC diagnosis*			
No	7 (21.2)	2 (3.6)	
Yes	26 (78.8)	53 (96.4)	0.01^2^
*History of SCC and BCC prior to index SCC diagnosis*			
No	7 (21.9)	1 (2.0)	0.005^2^
Yes	25 (78.1)	49 (98.0)	
*Number of HPV types in index SCC tumors*			
0	23 (42.6)	22 (28.6)	0.17
1	12 (22.2)	14 (18.2)	
2–5	17 (31.5)	33 (42.9)	
>5	2 (3.7)	8 (10.4)	

^1^Student's *t*-test. ^2^Fisher's exact test.

**Table 3:** Association of seropositivity to cutaneous human papillomavirus (HPV) types at the time of the diagnosis of index SCC and risk of subsequent squamous cell carcinoma (SCC) of the skin.

Viral infection at the time of the index SCC diagnosis	No subsequent SCC (*n* = 59) *n* (%)	Subsequent SCC (*n* = 91) *n* (%)	HR (95% CI)^*∗*^
*Any cutaneous HPV* ^1^			
Seronegative to 33 cutaneous HPV types	6 (10.2)	9 (9.9)	1.00
Seropositive to ≥1 cutaneous HPV	53 (89.8)	82 (90.1)	0.83 (0.41–1.67)
*Alpha cutaneous HPV*			
Seronegative to 33 cutaneous HPV types	6 (21.4)	9 (20.4)	1.00
Alpha HPV seropositive^2^	22 (78.6)	35 (79.6)	0.86 (0.40–1.82)
Alpha HPV seronegative	37 (62.7)	56 (61.5)	
Alpha HPV seropositive^3^	22 (37.3)	35 (38.5)	1.04 (0.68–1.59)
*Beta HPV*			
Seronegative to 33 cutaneous HPV types	6 (12.0)	9 (12.0)	1.00
Beta HPV seropositive^2^	44 (88.0)	66 (88.0)	0.80 (0.39–1.62)
Beta HPV seronegative	15 (25.4)	25 (27.5)	1.00
Beta HPV seropositive^3^	44 (74.6)	66 (72.5)	0.82 (0.51–1.30)
*Any beta 1 HPV* ^4^			
Seronegative	6 (17.6)	9 (16.4)	1.00
Beta 1 HPV seropositive	28 (82.3)	46 (83.6)	0.77 (0.37–1.59)
*Any beta 2 HPV* ^5^			
Seronegative	6 (17.6)	9 (15.0)	1.00
Beta 2 HPV seropositive	28 (82.3)	51 (85.0)	0.99 (0.48–2.04)
*Multiple beta 1 HPV* ^6^			
Seronegative	18 (52.9)	24 (43.6)	1.00
Seropositive to ≥2 beta 1 HPV	16 (47.1)	31 (56.4)	1.06 (0.61–1.83)
*Multiple beta 2 HPV* ^7^			
Seronegative	18 (52.9)	25 (41.7)	1.00
Seropositive to ≥2 beta 2 HPV	16 (47.1)	35 (58.3)	0.98 (0.58–1.67)
*Gamma HPV*			
Seronegative to 33 cutaneous HPV types	6 (15.0)	9 (13.2)	1.00
Seropositive^2^	34 (85.0)	59 (86.8)	0.93 (0.46–1.90)
Gamma HPV Seronegative	25 (42.4)	32 (35.2)	1.00
Seropositive^3^	34 (57.6)	59 (64.8)	1.21 (0.78–1.87)
*Mu HPV*			
Seronegative to 33 cutaneous HPV types	6 (22.2)	9 (20.5)	1.00
Seropositive^2^	21 (77.8)	35 (79.5)	0.78 (0.37–1.65)
Mu HPV seronegative	38 (64.4)	56 (61.5)	1.00
Seropositive^3^	21 (35.6)	35 (38.5)	1.05 (0.68–1.61)
*Nu HPV*			
Seronegative to 33 cutaneous HPV types	6 (66.7)	9 (39.1)	1.00
Seropositive^2^	3 (33.3)	14 (60.9)	0.96 (0.38–2.44)
Nu HPV seronegative	56 (94.9)	77 (84.6)	1.00
Seropositive^3^	3 (5.1)	14 (15.4)	1.31 (0.73–2.34)

^1^Seropositivity to any cutaneous HPV (*n* = 33 types) versus negative to all. ^2^Seropositive to at least one genus specific type versus negative to all cutaneous (*n* = 33) types. ^3^Seropositive to at least one genus specific HPV versus seronegative to all genus-specific HPV types. ^4^Seropositive to at least one beta 1 type versus seronegative to 33 cutaneous HPV types. ^5^Seropositive to at least one beta 2 type versus seronegative to 33 cutaneous HPV types. ^6^Seropositive to ≥2 beta 1 HPV types versus seronegative to all cutaneous types or positive to at least one beta 1 HPV type. ^7^Seropositive to ≥2 beta 2 HPV types versus seronegative to all cutaneous types or positive to at least one beta 2 HPV type. HR = hazards ratio, CI = confidence interval. ^*∗*^Adjusted for age and gender.

**Table 4:** Association between cutaneous human papillomavirus (HPV) infection in *eyebrow hair* and the risk of subsequent squamous cell carcinoma (SCC).

Eyebrow hair viral infection	No subsequent SCC (*n* = 59)	Subsequent SCC (*n* = 91)	^*∗*^HR (95% CI)
at the index SCC diagnosis			
*Any beta HPV* ^1^			
DNA negative	6 (10.5)	12 (13.6)	1.00
DNA positive	51 (89.5)	76 (86.4)	0.84 (0.45–1.56)
*Any beta 1 HPV* ^2^			
DNA negative	6 (13.6)	12 (16.2)	1.00
DNA positive	38 (86.4)	62 (83.8)	0.84 (0.44–1.58)
*Any beta 2 HPV* ^3^			
DNA negative	6 (12.0)	12 (16.2)	1.00
DNA positive	44 (88.0)	62 (83.8)	0.85 (0.45–1.59)
*Multiple beta 1 HPV* ^4^			
DNA negative	25 (56.8)	37 (50.0)	1.00
DNA positive	19 (43.2)	37 (50.0)	1.18 (0.72–1.93)
*Multiple beta 2 HPV* ^5^			
DNA negative	29 (58.0)	35 (47.3)	1.00
DNA positive	21 (42.0)	39 (52.7)	1.23 (0.75–2.01)

^1^Eyebrow hair DNA positive to any beta HPV type versus negative to all 25 beta HPV types. ^2^Eyebrow hair DNA positive to at least one beta 1 HPV type versus DNA negative to 25 beta HPV types. ^3^Eyebrow hair DNA positive to at least one beta 2 HPV type versus DNA negative to 25 beta HPV types. ^4^Eyebrow hair DNA positive to 2 or more beta-1 HPV types versus DNA negative to 25 beta HPV types or positive to one beta 1 HPV type. ^5^Eyebrow hair DNA positive to 2 or more beta 2 HPV types versus DNA negative to 25 beta HPV types or positive to one beta 2 HPV type. HR = hazards ratio, CI = confidence interval. ^*∗*^Adjusted for age and gender.

**Table 5:** Association of beta HPV infection in *eyebrow hair* and beta HPV *seropositivity* with the risk of subsequent SCC *by index tumor HPV DNA status*.

Viral infection at the time of index SCC diagnosis	HPV DNA negative index SCC			HPV DNA positive index SCC		
	No subsequent SCC (*n* = 23)	Subsequent SCC (*n* = 22)	HR (95% CI)	No subsequent SCC (*n* = 31)	Subsequent SCC (*n* = 55)	HR (95% CI)
HPV serology						
*Any beta HPV* ^1,3^						
Beta HPV seronegative	7 (30.4)	10 (45.5)	1.00	7 (22.6)	12 (21.8)	1.00
Beta HPV seropositive	16 (69.6)	12 (54.5)	0.44 (0.18–1.03)	24 (77.4)	43 (78.9)	0.92 (0.48–1.75)
HPV infection in eyebrow hairs						
*Any beta HPV* ^2,3^						
DNA negative to all beta HPV types	3 (13.6)	6 (27.3)	1.0	2 (6.7)	5 (9.4)	1.0
DNA positive to ≥1 beta HPV	19 (86.4)	16 (72.7)	0.97 (0.38–2.50)	28 (93.3)	48 (90.6)	**0.30 (0.11–0.78)**
*Any beta 1 HPV* ^4^						
DNA negative	3 (25.00)	6 (33.3)	1.0	2 (11.1)	1 (2.8)	1.0
DNA positive	9 (75.0)	12 (66.7)	1.21 (0.45–3.24)	16 (88.9)	35 (97.2)	0.72 (0.09–5.45)
*Any beta 2 HPV* ^4^						
DNA negative	3 (15.0)	6 (35.3)	1.0	1 (4.8)	5 (12.8)	1.0
DNA positive	17 (85.0)	11 (64.7)	0.85 (0.31–2.32)	20 (95.2)	34 (87.2)	**0.32 (0.12–0.86)**

^1^Any beta HPV seropositivity was defined as seropositive to ≥1 beta HPV types versus seronegative to all beta HPV types. ^2^Any beta HPV infection in eyebrow hairs was defined as DNA positivity to ≥1 beta HPV types versus DNA negativity to all beta HPV types. ^3^Tumor DNA status was defined as DNA positive to one or more beta HPV types versus DNA negative to all beta HPV types. ^4^Tumor DNA status was defined as DNA positivity to species-specific beta HPV types versus DNA negative to all beta HPV types.
